# Development and validation of a clinical predictive model for high-volume lymph node metastasis of papillary thyroid carcinoma

**DOI:** 10.1038/s41598-024-66304-6

**Published:** 2024-07-09

**Authors:** Hanlin Zhu, Haifeng Zhang, Peiying Wei, Tong Zhang, Chunfeng Hu, Huijun Cao, Zhijiang Han

**Affiliations:** 1 Department of Radiology, Hangzhou Ninth People’s Hospital, No. 98, Yilong Road, Qiantang District, Hangzhou, 310012 China; 2 Department of Radiology, Affiliated Hangzhou First People’s Hospital, Westlake University School of Medicine, No. 261, Huansha Road, Shangcheng District, Hangzhou, 310006 China

**Keywords:** Papillary thyroid carcinoma, Central lymph node metastasis, High volume, Clinical predictive model, Cancer, Surgical oncology, Thyroid diseases

## Abstract

The central lymph node metastasis (CLNM) status in the cervical region serves as a pivotal determinant for the extent of surgical intervention and prognosis in papillary thyroid carcinoma (PTC). This paper seeks to devise and validate a predictive model based on clinical parameters for the early anticipation of high-volume CLNM (hv-CLNM, > 5 nodes) in high-risk patients. A retrospective analysis of the pathological and clinical data of patients with PTC who underwent surgical treatment at Medical Centers A and B was conducted. The data from Center A was randomly divided into training and validation sets in an 8:2 ratio, with those from Center B serving as the test set. Multifactor logistic regression was harnessed in the training set to select variables and construct a predictive model. The generalization ability of the model was assessed in the validation and test sets. The model was evaluated through the receiver operating characteristic area under the curve (AUC) to predict the efficiency of hv-CLNM. The goodness of fit of the model was examined via the Brier verification technique. The incidence of hv-CLNM in 5897 PTC patients attained 4.8%. The occurrence rates in males and females were 9.4% (128/1365) and 3.4% (156/4532), respectively. Multifactor logistic regression unraveled male gender (OR = 2.17, *p* < .001), multifocality (OR = 4.06, *p* < .001), and lesion size (OR = 1.08 per increase of 1 mm, *p* < .001) as risk factors, while age emerged as a protective factor (OR = 0.95 per an increase of 1 year, *p* < .001). The model constructed with four predictive variables within the training set exhibited an AUC of 0.847 ([95%CI] 0.815–0.878). In the validation and test sets, the AUCs were 0.831 (0.783–0.879) and 0.845 (0.789–0.901), respectively, with Brier scores of 0.037, 0.041, and 0.056. Subgroup analysis unveiled AUCs for the prediction model in PTC lesion size groups (≤ 10 mm and > 10 mm) as 0.803 (0.757–0.85) and 0.747 (0.709–0.785), age groups (≤ 31 years and > 31 years) as 0.778 (0.720–0.881) and 0.837 (0.806–0.867), multifocal and solitary cases as 0.803 (0.767–0.838) and 0.809 (0.769–0.849), and Hashimoto’s thyroiditis (HT) and non-HT cases as 0.845 (0.793–0.897) and 0.845 (0.819–0.871). Male gender, multifocality, and larger lesion size are risk factors for hv-CLNM in PTC patients, whereas age serves as a protective factor. The clinical predictive model developed in this research facilitates the early identification of high-risk patients for hv-CLNM, thereby assisting physicians in more efficacious risk stratification management for PTC patients.

## Introduction

Papillary thyroid carcinoma (PTC) accounts for approximately 90% of primary malignant tumors in the thyroid gland, with 30% to 90% of patients diagnosed presenting with concurrent cervical lymph node metastasis^1,2^, particularly involving central lymph node metastasis (CLNM). The conventional treatment approach for PTC involves surgical excision with or without cervical lymph node dissection. While this method allows for the thorough removal of the primary tumor and metastatic foci, its limitations include an increased incidence of complications such as damage to the parathyroid glands and recurrent laryngeal nerves^3,4^. With the rising detection rates of PTC and an enhanced understanding of its biological behavior, there is an increasing body of research advocating for ultrasound-guided radiofrequency ablation or active clinical observation for low-risk PTC cases^5,6^. In comparison to surgical resection, these two approaches offer advantages in terms of minimizing trauma and economic burden. However, difficulties persist, particularly in the early identification of CLNM^7^, especially in cases of high-volume CLNM (hv-CLNM, > 5 nodes). The 2015 edition of the American Thyroid Association (ATA) management guidelines stipulate that the risk level for hv-CLNM in PTC patients is categorized as medium risk. Clearly, a comprehensive preoperative assessment of lymph node metastasis risk, coupled with the optimal timing for lymph node dissection, will enhance both the quality of life and survival rates for patients^8^. Consequently, regardless of the chosen therapeutic modality, pre-treatment risk stratification management for hv-CLNM in PTC patients is a crucial aspect.

Ultrasonography is the most commonly employed imaging modality for evaluating and monitoring cervical lymph node metastasis in PTC patients. Nevertheless, its efficacy in evaluating CLNM is limited by the presence of gas and bone, with sensitivity and accuracy ranging from 17 to 38% and 58% to 71%, respectively^9–11^. As opposed to ultrasonography, CT scans provide better visualization of the central lymph nodes; however, their sensitivity and accuracy are not superior, ranging from 23 to 50% and 55% to 75%, respectively^9–12^. Therefore, relying solely on typical imaging features from ultrasonography or CT for the direct diagnosis of CLNM in PTC patients does not meet clinical demands. Age, gender, lesion size, and Hashimoto’s thyroiditis (HT) status play crucial roles in forecasting hv-CLNM in PTC patients. Nomogram models based on clinical factors have received initial recognition^13,14^. Notwithstanding, these studies primarily originate from single medical centers, with sample sizes ranging from 729 to 2329 cases or focusing solely on specific subgroups of PTC. Currently, there is a lack of large-sample, bicentric or multicenter studies specifically addressing hv-CLNM in PTC patients.

Based on pathological and clinical data from 5897 cases of PTC across two medical centers, this work endeavors to construct a clinical predictive model for hv-CLNM in PTC patients. The aim is to explore the discriminative capabilities of the model within various subgroups, considering factors such as lesion size, lesion quantity, age, and the presence of HT. The objective is to furnish clinicians with valuable insights for predicting hv-CLNM in PTC patients, thereby enhancing support for clinical stratified management and proactive therapeutic interventions.

## Methods

### Ethics statement

This study was approved by the Ethics Committee of the First People’s Hospital affiliated with Westlake University and Hangzhou Cancer Hospital (Approval numbers: ZN-20230131-0014-01, HZCH-2023-008), and patient informed consent was waived. Furthermore, it adhered to the ethical standards set forth by the World Medical Association’s Declaration of Helsinki.

### Study population

**Figure 1:**
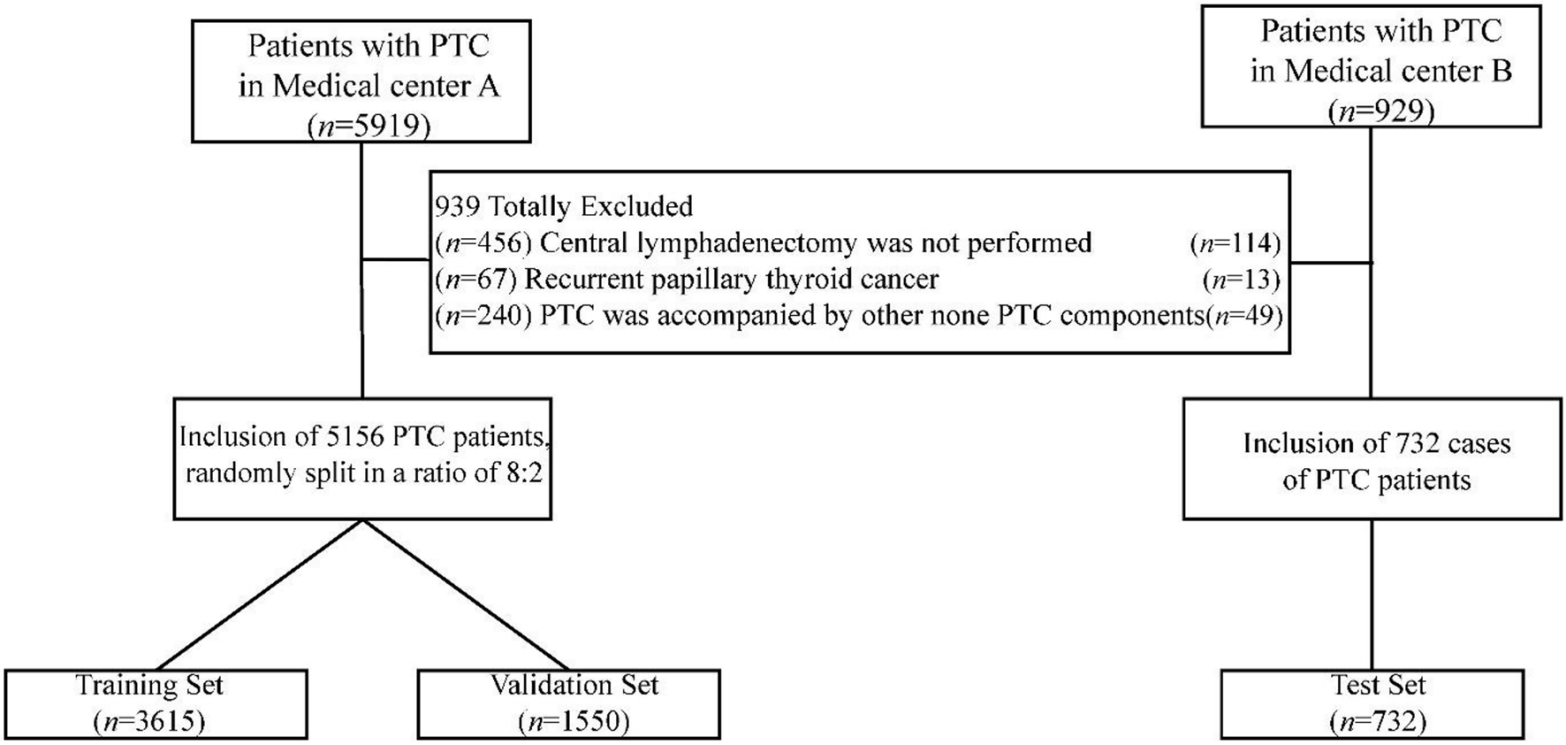
Flowchart of Training Set, Validation and Test Sets for PTC Patients’ hv-CLNM Predictive Model. Note: PTC, papillary thyroid carcinoma; hv-CLNM, high-volume central lymph node metastasis.

The clinical data of patients who underwent surgery for PTC from January 2010 to August 2023 at the Hangzhou First People’s Hospital affiliated to Westlake University Medical School (Medical Center A) and Hangzhou Cancer Hospital (Medical Center B) were retrospectively collected. The collected data encompassed gender, age, maximum lesion size, lesion quantity and location, as well as the coexistence of HT. Cases were excluded based on predefined criteria: (1) absence of central lymph node dissection; (2) recurrent thyroid cancer; (3) The concurrent presence of other malignant cervical tumors, such as squamous cell carcinoma, lymphoma, other malignancies of the thyroid (medullary carcinoma, follicular carcinoma, etc.), and subtypes of non-classic papillary thyroid carcinoma (Tall cell subtype, Diffuse sclerosing subtype, etc.); (4) patients who underwent neck radiation or systemic chemotherapy (Fig. 1).

### Surgical interventions

(1) Unilateral lobectomy plus isthmectomy + central lymph node dissection; (2) Total thyroidectomy + central lymph node dissection, followed by the consideration of lateral neck lymph node dissection based on clinical and imaging examination results in patients.

### Variable collection and outcome measures

In the clinical and pathological electronic systems, a keyword search for “papillary thyroid carcinoma” was conducted to retrieve pathological outcomes. Patient hospital records were then reviewed to harvest data including age, gender, coexistence of HT, number of lesions, maximum diameter and location of lesions, and CLNM status. On the basis of the quantity of CLNM, cases were categorized into the high-volume metastasis group (> 5 nodes) and the low-volume metastasis group^7^.

### Model construction and statistical analysis

The data from Medical Center A was randomly partitioned into training and validation sets in an 8:2 ratio, whereas the data from Medical Center B was designated as the test set. Within the training set, single-factor logistic regression analysis was utilized to screen variables, retaining those with *p*-values < 0.05. Subsequently, utilizing multifactor regression analysis, independent risk factors predicting hv-CLNM were identified. This process led to the establishment of a clinical predictive model, which was then validated for diagnostic performance in the validation and test sets. The discriminative capacity of the model was examined through metrics including the receiver operating characteristic area under the curve (AUC), sensitivity, specificity, accuracy, positive likelihood ratio (PL +), and negative likelihood ratio (NL-). Calibration curves evaluated the calibration accuracy of the model, while the Brier score quantified its calibration precision. Clinical decision curve analysis (DCA) was introduced to confirm the clinical applicability of the model.

Descriptive continuous data were expressed using medians and interquartile range (IQR), while categorical variables were represented as component ratios (percentages). Restricted cubic splines (RCS, with linear restriction point *n* = 3) were adopted to capture the non-linear relationships between age, PTC lesion size, and the occurrence of hv-CLNM within the model. A significance level of *p* < 0.05 was employed to denote statistically significant differences. All analyses were conducted with the use of Python software (version 3.7.1, Python Software Foundation, Delaware, USA) and R software (version 4.0.1; R Foundation for Statistical Computing, Vienna, Austria). An online hv-CLNM predictive model calculator for PTC patients was established and is available at https://lgh384712.shinyapps.io/hv-CLNM-model/.

## Results

### Characteristics of study patients

Medical Center A incorporated a total of 5165 patients with PTC, with males constituting 22.8% (1176/5165) and females 77.2% (3989/5165). The median age was 47 years ([IQR] 37–55), and the incidence of hv-CLNM was 4.5% (233/5165). Medical Center B enrolled 732 PTC patients, with males and females comprising 25.8% (189/732) and 74.2% (543/732), respectively. The median age was 48 years (39–55), and the hv-CLNM incidence attained 7.0% (51/732). Table 1 provides details of the randomly split dataset and other clinical characteristics.

**Table 1:** Clinicopathological characteristics of patients with PTC in two medical centers.

Characteristic	Total (*n* = 5897)	Training Set (*n* = 3615)	Validation Set (*n* = 1550)	Test Set (*n* = 732)
Age, median (IQR), y	47 (37–55)	47 (37–55)	47 (36–55)	48 (39–55)
Gender, No. (%)				
Female	4532 (76.9)	2786 (77.1)	1203 (77.6)	543 (74.2)
Male	1365 (23.1)	829 (22.9)	347 (22.4)	189 (25.8)
Location, No. (%)				
Right	2452 (41.6)	1357 (37.5)	609 (39.3)	421 (57.5)
Left	2221 (37.7)	1500 (41.5)	617 (39.8)	104 (14.2)
Bilateral	703 (11.9)	394 (10.9)	169 (10.9)	140 (19.1)
Isthmus	521 (8.8)	364 (10.1)	155 (10.0)	67 (9.2)
Multifocality, No. (%)				
No	4613 (78.2)	2900 (80.2)	1215 (78.4)	498 (68.0)
Yes	1284 (21.8)	715 (19.8)	335 (21.6)	234 (32.0)
Size, Median (IQR), mm	6.0 (4.0–10.0)	6.0 (4.0–10.0)	6.0 (4.0–10.0)	7.0 (4.0–11.0)
Subgrouping by size, No. (%)				
≤ 10 mm	4629 (78.5)	2867 (79.3)	1226 (79.1)	536 (73.2)
> 10 mm	1268 (21.5)	748 (20.7)	324 (20.9)	196 (26.8)
hv-CLNM, No. (%)				
Negative	5613 (95.2)	3458 (95.7)	1474 (95.1)	681 (93.0)
Positive	284 (4.8)	157 (4.3)	76 (4.9)	51 (7.0)
HT, No. (%)				
Negative	4636 (78.6)	2799 (77.4)	1190 (76.8)	647 (88.4)
Positive	1261 (21.4)	816 (22.6)	360 (23.2)	85 (11.6)

PTC, papillary thyroid carcinoma; CLNM, central lymph node metastasis; hv-CLNM, high-volume CLNM; HT, Hashimoto’s thyroiditis.

### Variable selection in clinical prediction model

As suggested by the single-factor analysis within the training set, male gender (OR = 2.63), larger lesion size (OR = 1.11 per increase of 1 mm), multifocality (OR = 5.02), and bilateral occurrence (OR = 5.48) all emerged as risk factors, while age (OR = 0.95 per increase of 1 year) served as a protective factor (all *p*-values < 0.001), with no statistically significant disparities observed in the coexistence of HT (*p* = 0.148). The outcomes of the multiple-factor analysis denoted that male gender (OR = 2.17), lesion size (OR = 1.08), multifocality (OR = 4.06), and age (OR = 0.95) continued to display statistical differences (all *p*-values < 0.001), whereas bilateral occurrence showed no statistically significant difference (*p* = 0.309) (Table 2).

**Table 2:** Training set for single-factor and multiple-factor logistic regression analysis related to PTC hv-CLNM.

		Single-factor		Multiple-factor	
		OR (95%CI)	*p*	OR (95%CI)	*p*
Gender	Female	(reference)		(reference)	
	Male	2.63 (1.90–3.65)	< .001	2.17 (1.50–3.14)	< .001
Age	Age	0.95 (0.93–0.96)	< .001	0.95 (0.94–0.97)	< .001
Location	Right	(reference)		(reference)	
	Left	1.24 (0.81–1.88)	.320	1.29 (0.83–2.01)	.262
	Bilateral	5.48 (3.58–8.41)	< .001	1.36 (0.75–2.45)	.309
	Isthmus	0.95 (0.47–1.93)	.897	1.21 (0.57–2.60)	.621
Multifocality	No	(reference)		(reference)	
	Yes	5.02 (3.62–6.94)	< .001	4.06 (2.46–6.72)	< .001
Size (mm)		1.11 (1.09–1.12)	< .001	1.08 (1.06–1.10)	< .001
HT	Negative	(reference)		(reference)	
	Positive	0.74 (0.49–1.12)	.148	0.69 (0.44–1.09)	.109

PTC, papillary thyroid carcinoma; CI, confidence interval; HT, Hashimoto thyroiditis; hv-CLNM, high-volume central lymph node metastasis.

### Diagnostic performance of the clinical prediction model

**Figure 2:**
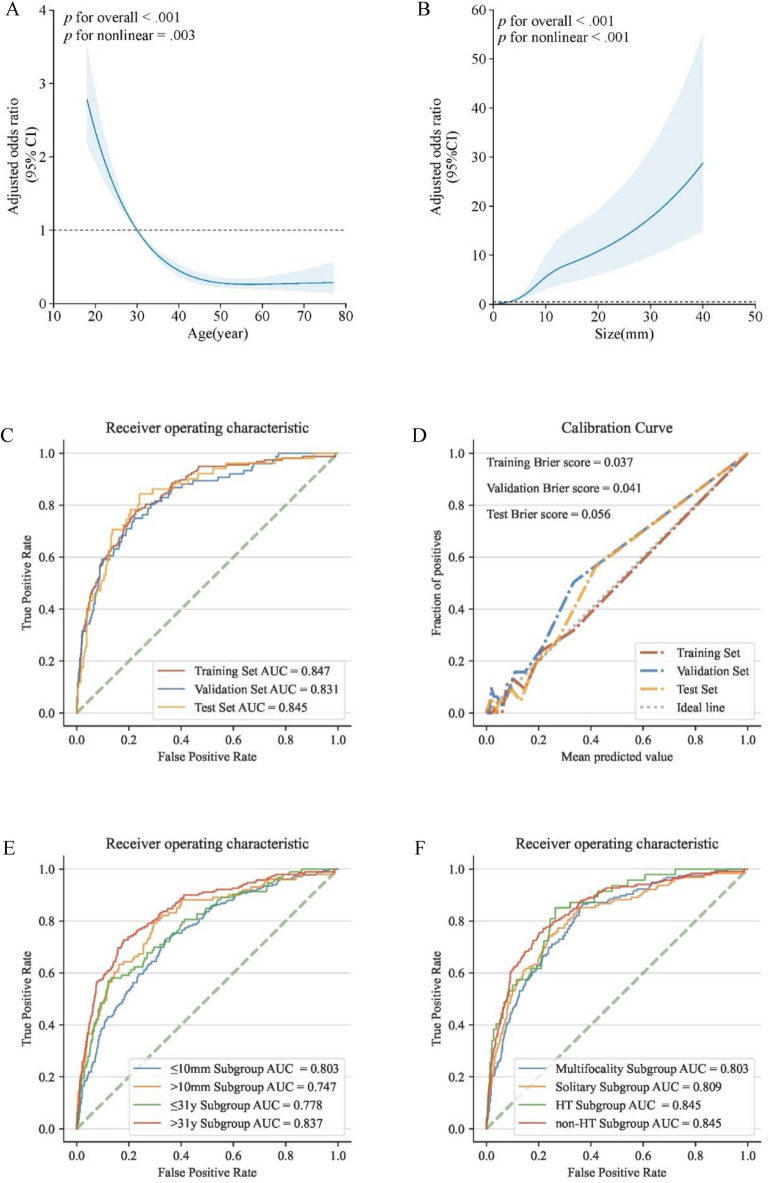
Presents RCS analysis, where the shaded areas represent the 95%CI derived from the RCS model. OR values for age (**A**) and lesion size (**B**) were adjusted based on other variables in the model. The ROC analysis (**C**) and calibration curves (**D**) for the clinical predictive model of PTC patients with hv-CLNM are depicted across three datasets. Additionally, ROC analyses for the clinical predictive model are displayed within eight subgroups (E–F). Note: RCS, restricted cubic spline; AUC, area under the curve; ROC, receiver operating characteristic curve; hv-CLNM, high-volume central lymph node metastasis.

The results of the model fitted with RCS curves confirmed, after confounding factors were adjusted, a certain degree of non-linear decreasing relationship between age and the risk of hv-CLNM (*p* for nonlinear = 0.003). Within the cohort aged ≤ 31 years, age acted as a risk factor (OR > 1); nonetheless, beyond this age threshold, it transitioned to a protective factor (OR < 1) (Fig. 2A). The risk of hv-CLNM occurrence was relatively low when the lesion size was < 5 mm, with a rapid increase in the OR value observed after the lesion diameter surpassed this size threshold (*p* for non-linearity < 0.001) (Fig. 2B).

Based on gender, age, lesion size, and multifocality, a predictive model for hv-CLNM was constructed. As depicted in Fig. 2C, the model exhibited an AUC of 0.847 (95% CI 0.815–0.878) in the training set, with corresponding sensitivity and specificity values of 78.3% and 77.8%, respectively. In both validation and test sets, the AUCs stood at 0.831 (0.783–0.879) and 0.845 (0.789–0.901), with sensitivities of 78.9% and 82.4%, and specificities of 78.3% and 77.2%, respectively (Table 3). Calibration curve outcomes displayed a high degree of consistency between the clinical model predictions and actual observations across all three datasets, with Brier scores of 0.037, 0.041, and 0.056, all less than 0.1 (Fig. 2D). DCA findings indicated that the model surpassed both the baseline of treating all or treating none for most threshold ranges (Supplementary Fig. 1).

**Table 3:** The clinical predictive model predicts the performance of hv-CLNM in PTC patients.

	AUC	Sensitivity (%)	Specificity (%)	Accuracy (%)	LR +	LR-
Training Set	0.847 (0.815–0.878)	78.3 (70.1–91.1)	77.8 (62.7–82.9)	77.9 (63.9–82.5)	3.537 (1.88–5.338)	0.278 (0.477–0.108)
Validation Set	0.831 (0.783–0.879)	78.9 (61.8–90.8)	78.3 (63.1–91.1)	78.3 (64.3–89.8)	3.637 (1.676–10.216)	0.269 (0.605–0.101)
Test Set	0.845 (0.789–0.901)	82.4 (68.6 − 94.1)	77.2 (71.2 − 88.0)	77.9 (72.3–87.2)	3.618 (2.384–7.816)	0.228 (0.441–0.067)

AUC, area under the roc curve; The parentheses indicate the 95% confidence interval; LR + , Positive Likelihood Ratio; LR-, Negative Likelihood Ratio; hv-CLNM, high-volume central lymph node metastasis.

### Subgroup analysis

The discriminative capability of the hv-CLNM clinical predictive model was slightly higher in the group with lesion size ≤ 10 mm compared to the > 10 mm group, with AUCs of 0.803 (0.757–0.850) and 0.747 (0.709–0.785), respectively. Based on the intersection point of the main line of RCS and the reference line where the OR equals 1, thresholding at the age of 31 years divided the cohort into two subgroups. The model’s predictive ability was higher in the > 31 years subgroup compared to the ≤ 31 years subgroup, with AUCs of 0.837 (0.806–0.867) and 0.778 (0.726–0.829), respectively (Fig. 2E). Within the multifocal and solitary subgroups, the model’s predictive performances were comparable, with AUCs of 0.803 (0.767–0.838) and 0.809 (0.769–0.849), respectively. Within the HT and non-HT subgroups, the model exhibited identical AUCs of 0.845, with only slight differences in the 95%CI (0.793–0.897 and 0.819–0.871) (Fig. 2F). Further details on the diagnostic performance of the model are provided in Supplementary Table 1. In terms of individual variables’ diagnostic efficiency, lesion size had the highest AUCs across all three datasets, ranging from 0.728 to 0.807. The AUC ranges for gender, age, and multifocality were 0.603–0.646, 0.636–0.674, and 0.644–0.684, respectively (see Supplementary Table 2 and Fig. 2).

## Discussion

The incidence of PTC has risen over the past two decades due to advancements in imaging diagnostic technologies, such as ultrasound and the increased utilization of fine needle aspiration biopsy^15^. While the prognosis for most PTC patients is favorable, some low-risk individuals still face a higher risk of recurrence or distant metastasis^16^, particularly those with hv-CLNM, where the risk of recurrence is five times higher than low-volume metastasis, and it is significantly associated with distant metastasis^17,18^. This underscores the importance of early identification and management of hv-CLNM. This study incorporated clinical and pathological data from 5897 PTC patients from two medical centers. The incidence of hv-CLNM was 9.4% (128/1365) in males and 3.4% (156/4532) in females. The clinical predictive model, built based on gender, age, lesion size, and multifocality, effectively identified high-risk PTC patients with hv-CLNM. The difference value in AUC between the validation and test sets (less than 0.05) suggested strong generalizability of the model. The model demonstrated consistent diagnostic performance across various subgroups (e.g., age groups, gender, multifocality, solitary, HT and non-HT), with AUC ranging from 0.747 to 0.837. These findings provide crucial insights for the clinical stratification management of PTC patients.

Young age is an independent risk factor for hv-CLNM in PTC patients, yet there is ongoing debate regarding the age threshold. Multiple studies have proposed age thresholds at 40 years^13,19–21^, 45 years^22,23^, or 55 years, with sample sizes ranging from 690 to 2363 cases. The outcomes corroborated an elevated risk of hv-CLNM in PTC patients aged ≤ 40 years or < 45 years, with OR ranging from 2.03 to 3.43. Li et al.’s research^14^ using 55 years as the threshold identified being < 55 years as a remarkable predictor for hv-CLNM, with a regression coefficient of 2.78. This research analyzed 5897 cases of PTC and confirmed younger age as an independent risk factor for hv-CLNM. In contrast to preceding studies, we refrained from categorizing age and instead incorporated it as a continuous variable into the model. This marks the inaugural utilization of RCS to investigate the non-linear correlation between age and hv-CLNM. Upon adjusting for confounding factors, we discovered that in PTC patients aged ≤ 31 years, the relative risk of hv-CLNM increased with each additional year, while beyond the age of 31 years, the risk gradually decreased. This hints a certain non-linear association between age and hv-CLNM. Although age categorization is commonplace in medical practice, some perspectives posit that this approach may not fully harness all available information^8^, potentially leading to misleading conclusions in clinical predictive models^24^. Therefore, this work treated age as a continuous variable. The findings demonstrated superior predictive performance of the model in the subgroup aged > 31 years compared to those aged ≤ 31 years. The intricate non-linear association between age and hv-CLNM might influence the diagnostic efficacy of the model within specific age subgroups. This discovery offers crucial insights for future research and clinical applications.

Gender is an indispensable predictive factor for hv-CLNM in PTC patients. Studies have unraveled that male patients display higher rates of CLNM, distant metastasis, and mortality compared to females^25^, with OR ranging from 2.02 to 3.39^23,26,27^. In our work, male gender emerged as an independent risk factor for hv-CLNM, with an OR value of 2.17. Despite the lower end of the literature-reported range, the substantial sample size mitigates statistical bias, providing a more accurate reflection of the true OR effect.

Lesion size is crucial for assessing the biological characteristics of PTC, with larger tumor sizes corresponding to higher risks of hv-CLNM. Most studies convert size into categorical variables, potentially resulting in information loss. Only Huang et al.’s study^23^, treating lesion size as a continuous variable, found an OR of 1.07 for each 10% increase. In our research, the OR for each 1 mm increase was 1.08. Interestingly, in subgroup analysis, our model demonstrated a higher AUC in forecasting PTC lesion sizes ≤ 10 mm compared to those > 10 mm. This suggests that the model is more adept at identifying high-risk hv-CLNM patients in low-risk PTC cases.

The correlation between multifocality and lymph node metastasis in PTC remains controversial. While some, like Geron et al.^28^, argue that multifocality is not an independent risk factor for CLNM, the majority of scholars consider it an independent risk factor for both CLNM and hv-CLNM, with OR ranging from 1.61 to 3.28^13,19–21^. In our dataset, the OR for multifocality was 4.06, and the hv-CLNM incidence (12.1%) was dramatically higher than that in the solitary lesion group (2.8%). The mechanism underlying the propensity of multifocal PTC to metastasize remains unclear, but some scholars lend support to the monoclonal origin theory^29^. This theory proposes that multiple cancer foci originate from the same tumor cells, and the primary lesion disseminates through the glandular lymphatic network, giving rise to multifocal lesions. This theory contributes to explaining the correlation between multifocal PTC and hv-CLNM. HT and lesion location showed no statistically significant divergence in this dataset.

The current research on clinical models predicting hv-CLNM in PTC is relatively limited. Wei et al.^30^ investigated 2395 cases of PTC, with an hv-CLNM incidence of 10.7%. Their predictive model demonstrated AUCs of 0.702 and 0.811 in the training and validation sets, respectively, but lacked diagnostic stability. Preliminary applications of deep learning in predicting PTC lymph node metastasis have shown promise. For instance, Yu et al.^31^ utilized a transfer learning radiomics model based on B-mode ultrasound image, achieving AUCs between 0.840 and 0.930. Nevertheless, the “black-box” nature of deep learning diminishes clinical interpretability. The model developed in this study, based on four easily accessible clinical variables, exhibited an AUC of 0.845 in the test set, offering the advantage of simplicity and strong clinical interpretability. While these two model approaches and technologies differ, they can complement each other. Both focus on forecasting PTC lymph node metastasis, differing only in their methods and data sources. Simple variables and high-dimensional variables each have their strengths and weaknesses, making it possible to choose between them or integrate both models for a comprehensive analysis based on the clinical context.

Limitations of this study encompass: (1) The retrospective design is inherently prone to selection and statistical biases; (2) Thyroid peroxidase antibody (TPOA) and thyroglobulin antibody (TGA) are commonly used laboratory indicators for clinically diagnosing HT. In this dataset, pathology served as the basis for diagnosing HT, and as such, TPOA and TGA were not included. Additionally, the correlation between TPOA and TGA may lead to model overfitting; (3) The clinical predictive model is based on a regional study of the northern Zhejiang population in China, and further multicenter studies are needed to determine its applicability in other countries and regions; (4) extrathyroidal extension had high levels of missingness and so were not imputed. Analyses incorporating those biomarkers were by complete-case analysis only, with the specific research findings detailed in Supplementary Material, Fig. 1, and Table 2.

In summary, male gender, larger lesion size, and multifocality emerge as independent risk factors for hv-CLNM in PTC patients. Age exhibits a negatively non-linear association with hv-CLNM, gradually transitioning into a protective factor with increasing age. The clinical predictive model developed in this study demonstrates the capability to identify high-risk PTC patients with hv-CLNM at an early stage, offering more robust support for clinical stratification management and proactive treatment.

### Supplementary Information

Supplementary Information.

## Data Availability

The datasets analyzed during the current study are not publicly available due to patient privacy concerns, but are available from the corresponding author on reasonable request.
