# Supplementary material for: Development and validation of a clinical predictive model for high-volume lymph node metastasis of papillary thyroid carcinoma
Source: Sci Rep. 2024 Jul 9;14:15828. doi: 10.1038/s41598-024-66304-6 (PMC11233634; doi:10.1038/s41598-024-66304-6)
Supplement: Supplementary file 1 — Supplementary Information. [file 41598_2024_66304_MOESM1_ESM.docx]

# Supplementary Materials

**Development and Validation of a Clinical Predictive Model for High-volume Lymph Node Metastasis of Papillary Thyroid Carcinoma**

Hanlin Zhu ^1,2^, Haifeng Zhang^2^, Peiying Wei ^2^, Tong Zhang ^2^, Chunfeng Hu ^2^, Huijun Cao^2^, Zhijiang Han ^2*^

*^1^. Department of Radiology, Hangzhou Ninth People’s Hospital, Hangzhou 310012, China*

*^2^. Department of Radiology, Affiliated Hangzhou First People's Hospital, Westlake University School of Medicine, Hangzhou 310006, China*

*. corresponding author.

| Supplementary table1 the diagnostic performance of PTC hv-CLNM clinical prediction models in subgroups | | | | | | | |
| --- | --- | --- | --- | --- | --- | --- | --- |
| subgroups | AUC | Sensitivity (%) | Specificity (%) | Accuracy (%) | LR+ | LR- |  |
| ≤10mm | 0.803  (0.757-0.85) | 82.2  (62.4-92.1) | 69.7  (59.2-86.1) | 69.9  (59.9-85.6) | 2.708  (1.529-6.608) | 0.256  (0.635-0.092) |  |
| >10mm | 0.747  (0.709-0.785) | 73.2  (56.8-82.0) | 66.4  (58.8-80.7) | 67.4  (61.4-77.4) | 2.177  (1.379-4.256) | 0.403  (0.734-0.223) |  |
| ≤31y | 0.778  (0.726-0.829) | 60.2  (50.5-85.0) | 87.2  (58.8-90.2) | 83.7  (61.7-86.5) | 4.703  (1.228-8.71) | 0.456  (0.841-0.167) |  |
| >31y | 0.837  (0.806-0.867) | 73.8  (66.5-84.8) | 82.0  (67.8-85.0) | 81.6  (68.5-84.5) | 4.093  (2.068-5.652) | 0.319  (0.494-0.178) |  |
| Multifocality | 0.803  (0.767-0.838) | 85.9  (69.9-91.7) | 64.3  (60.7-79.6) | 67.0  (63.9-78.6) | 2.404  (1.779-4.496) | 0.219  (0.496-0.105) |  |
| Solitary | 0.809  (0.769-0.849) | 80.5  (63.3-89.9) | 72.6  (64.0-86.3) | 72.8  (64.5-85.7) | 2.934  (1.756-6.554) | 0.269  (0.574-0.117) |  |
| HT | 0.845  (0.793-0.897) | 85.1  (72.3-95.7) | 74.1  (67.0-79.4) | 74.5  (67.8-79.3) | 3.28  (2.19-4.65) | 0.201  (0.413-0.054) |  |
| Non-HT | 0.845  (0.819-0.871) | 76.8  (66.7-86.1) | 79.4  (68.5-88.5) | 79.2  (69.5-87.4) | 3.725  (2.116-7.498) | 0.292  (0.487-0.157) |  |
| Note: PTC, Papillary thyroid carcinoma; hv-CLNM, high-volume central lymph node metastasis; HT, Hashimoto's thyroiditis. HT, Hashimoto's thyroiditis; ≤10mm, Papillary thyroid cancer lesion size ≤10mm; ＞10mm, Papillary thyroid cancer lesion size >10mm; ≤31y, the patient's age is ≤31 years old; >31y, the patient's age is >31 years old AUC, area under the roc curve; The parentheses indicate the 95% confidence interval; LR+, Positive Likelihood Ratio; LR-, Negative Likelihood Ratio | | | | | | | |

| Supplementary Table 2: Assessment of Diagnostic Performance for Gender, Age, Multifocality, and Tumor Size across the Training Set, Validation Set, and Test Set. | | | | | | | |
| --- | --- | --- | --- | --- | --- | --- | --- |
| Dataset and Variables | AUC | Sensitivity (%) | Specificity (%) | Accuracy (%) | LR+ | LR- |  |
| Training set |  |  |  |  |  |  |  |
| Gender | 0.603  (0.564-0.643) | 42.7  (35.0-50.3) | 77.9  (76.6-79.4) | 76.4  (75.0-77.8) | 1.934  (1.496-2.437) | 0.736  (0.848-0.626) |  |
| Age | 0.674  (0.628-0.72) | 59.9  (49.0-75.8) | 72.2  (52.9-77.5) | 71.7  (53.9-76.6) | 2.154  (1.042-3.373) | 0.556  (0.963-0.312) |  |
| Multifocality | 0.673  (0.633-0.713) | 52.9  (44.6-60.5) | 81.8  (80.5-83.1) | 80.5  (79.3-81.8) | 2.897  (2.284-3.577) | 0.577  (0.689-0.475) |  |
| Size | 0.807  (0.771-0.842) | 74.5  (64.3-85.4) | 75.5  (63.9-82.8) | 75.5  (64.9-82.2) | 3.039  (1.784-4.961) | 0.338  (0.558-0.177) |  |
| Validation Set |  |  |  |  |  |  |  |
| Gender | 0.646  (0.575-0.717) | 52.9  (39.2-66.7) | 76.2  (73.1-79.3) | 74.6  (71.4-77.6) | 2.225  (1.459-3.22) | 0.617  (0.831-0.42) |  |
| Age | 0.636  (0.545-0.726) | 62.7  (31.4-80.4) | 71.2  (50.4-92.5) | 70.1  (52.0-88.5) | 2.18  (0.632-10.735) | 0.523  (1.363-0.212) |  |
| Multifocality | 0.644  (0.574-0.715) | 58.8  (45.1-72.5) | 70.0  (66.5-73.6) | 69.3  (66.0-72.7) | 1.964  (1.347-2.745) | 0.588  (0.825-0.373) |  |
| Size | 0.728  (0.65-0.807) | 64.7  (43.1-86.3) | 76.4  (44.8-90.8) | 75.4  (48.1-87.7) | 2.737  (0.781-9.33) | 0.462  (1.27-0.151) |  |
| Test set |  |  |  |  |  |  |  |
| Gender | 0.618  (0.56-0.675) | 44.7  (34.2-56.6) | 78.8  (76.6-80.7) | 77.1  (75.0-79.1) | 2.107  (1.462-2.937) | 0.702  (0.859-0.538) |  |
| Age | 0.672  (0.609-0.734) | 48.7  (35.5-96.1) | 80.3  (28.3-87.9) | 78.8  (31.6-85.5) | 2.475  (0.495-7.954) | 0.639  (2.279-0.045) |  |
| Multifocality | 0.684  (0.627-0.741) | 56.6  (44.7-67.1) | 80.2  (78.2-82.2) | 79.0  (77.0-80.9) | 2.856  (2.048-3.761) | 0.541  (0.707-0.4) |  |
| Size | 0.783  (0.735-0.832) | 63.2  (75.0-89.5) | 55.2  (72.8-76.9) | 56.7  (72.8-76.8) | 2.757  (1.408-3.879) | 0.343  (0.668-0.137) |  |
| Note: AUC, area under the roc curve; The parentheses indicate the 95% confidence interval; LR+, Positive Likelihood Ratio; LR-, Negative Likelihood Ratio | | | | | | | |

| A  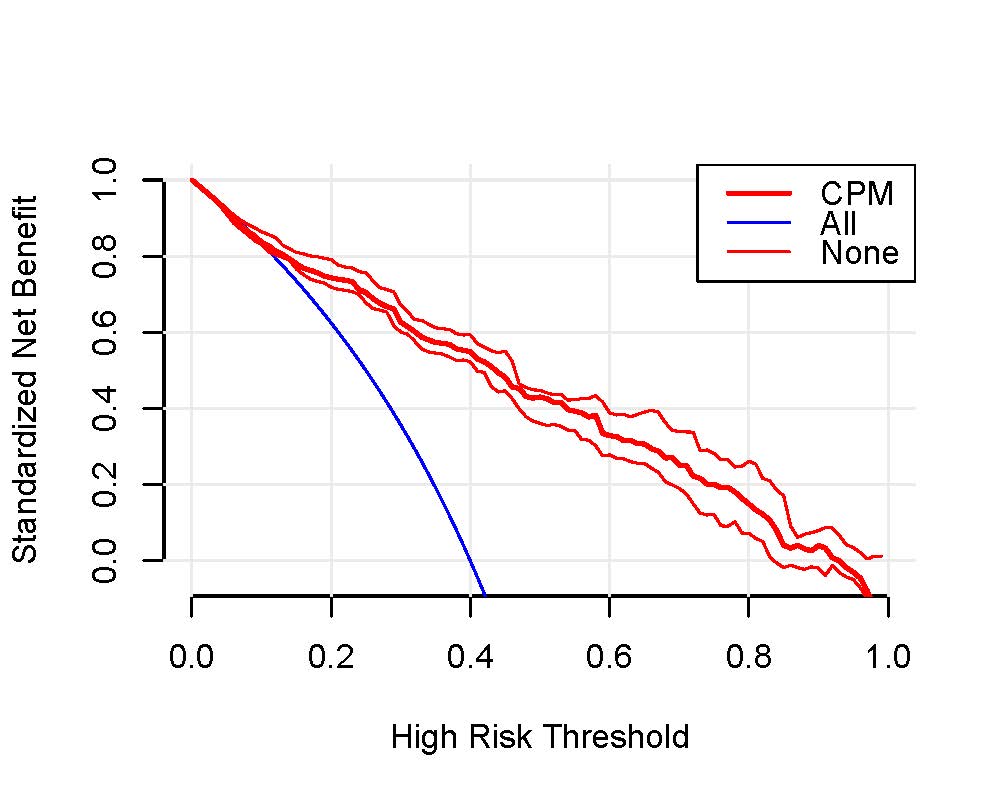 | B  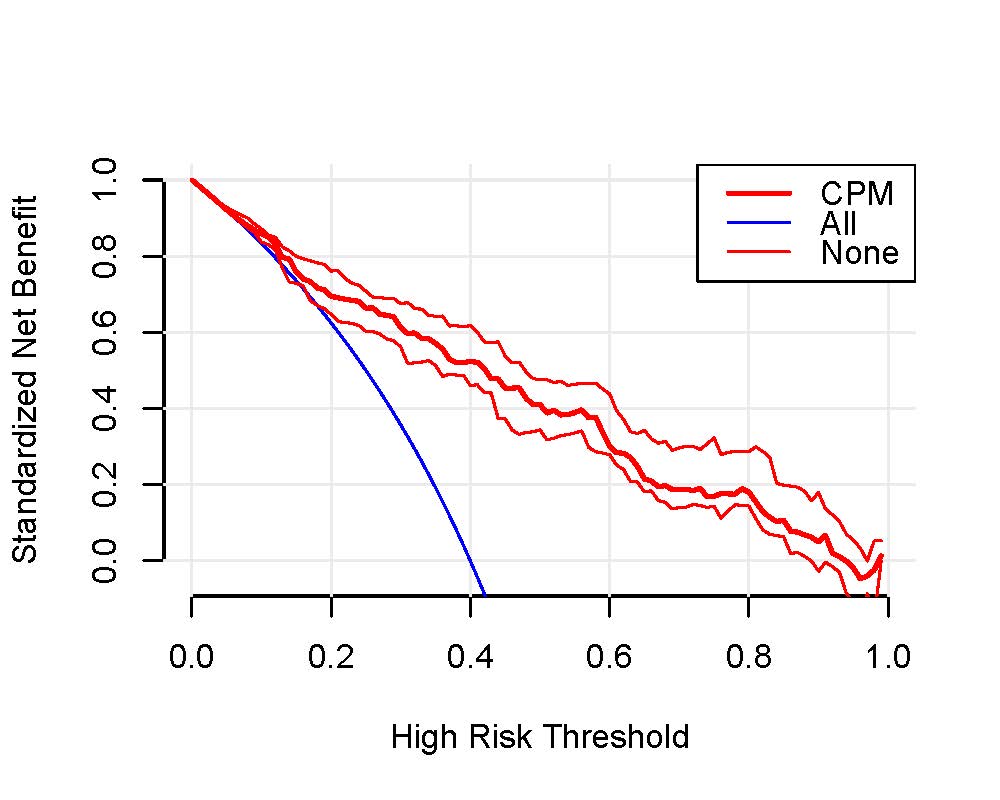 |
| --- | --- |
| C  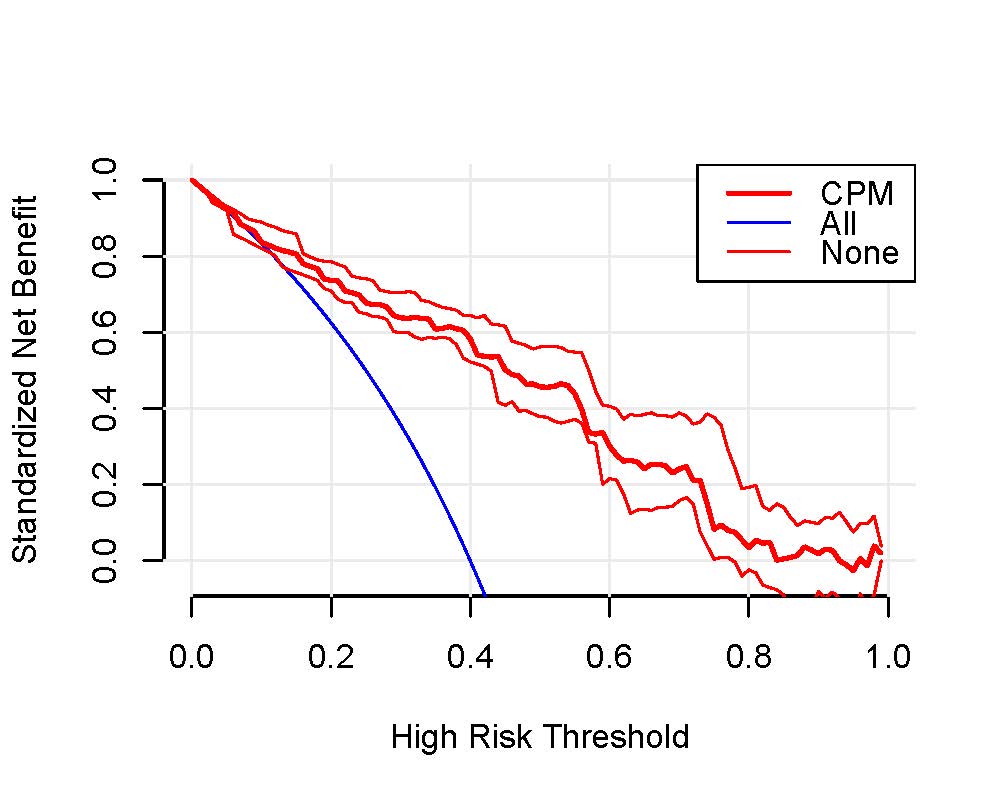 |  |

Supplementary Figure 1: Clinical decision curves for the PTC hv-CLNM clinical prediction model in the Training Set (A), Validation Set (B), and Test Set (C); with fine lines between representing 95% confidence intervals

Note: CPM , Clinical Prediction Model.

| A  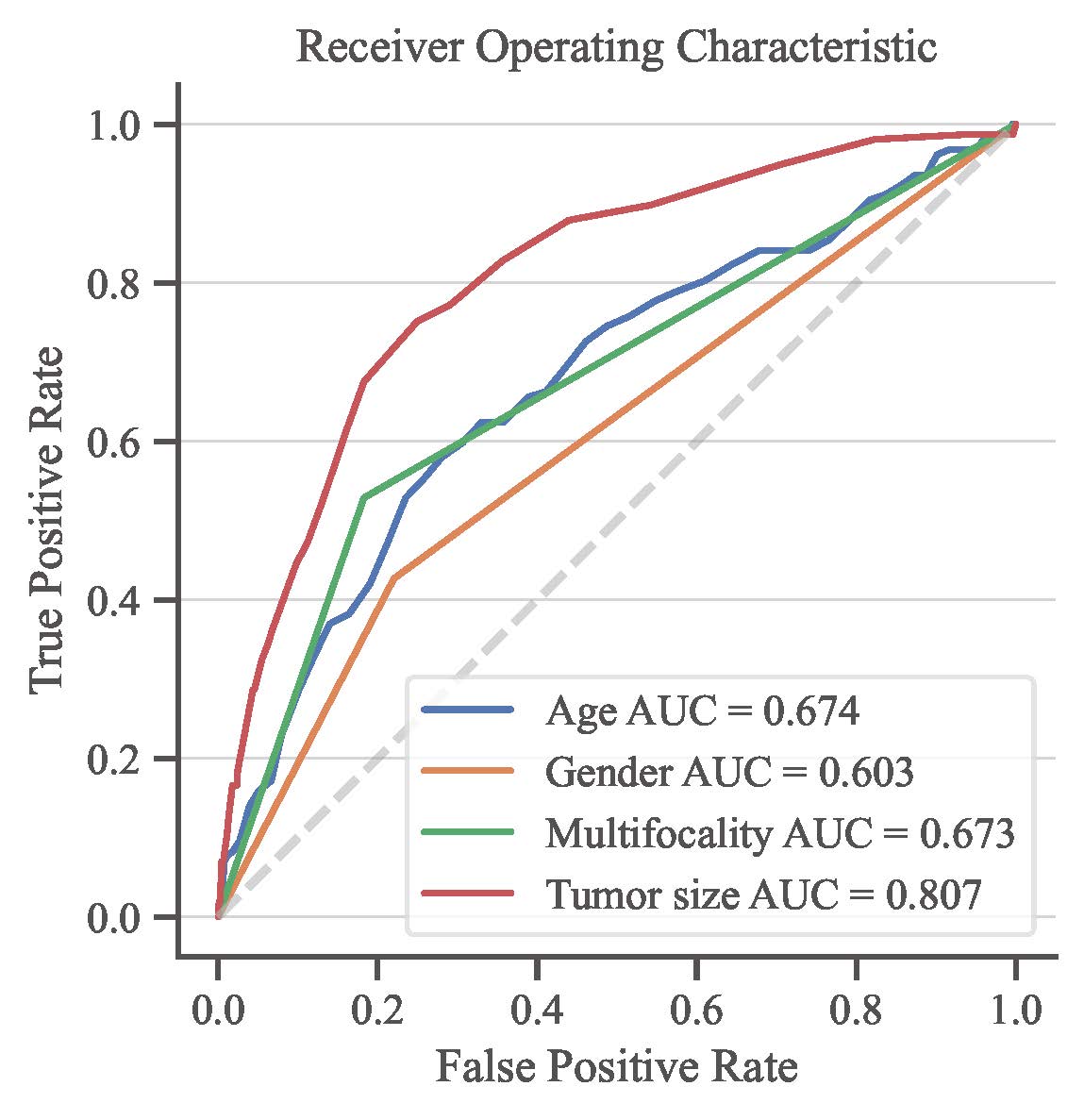 | B  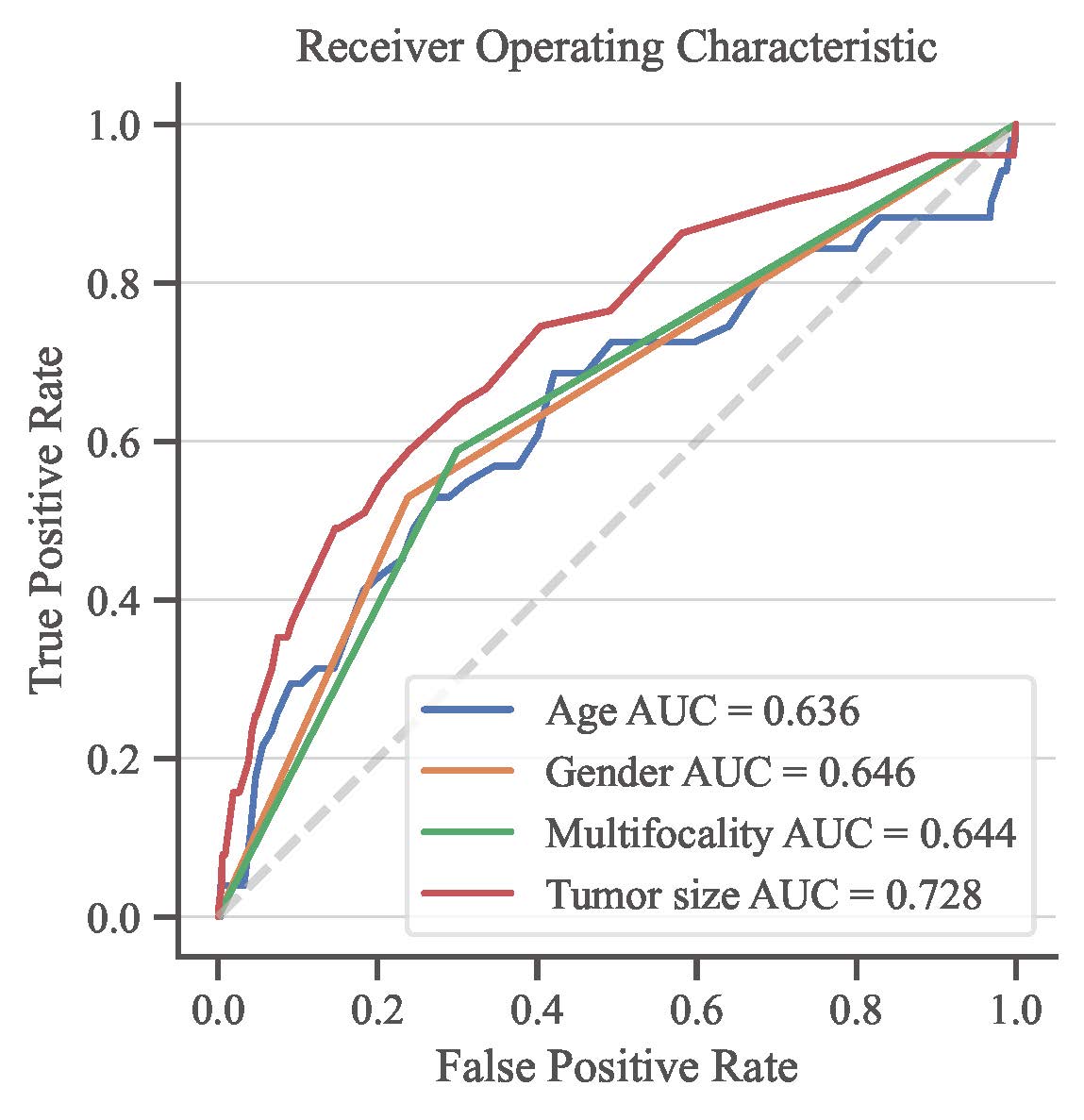 |
| --- | --- |
| C  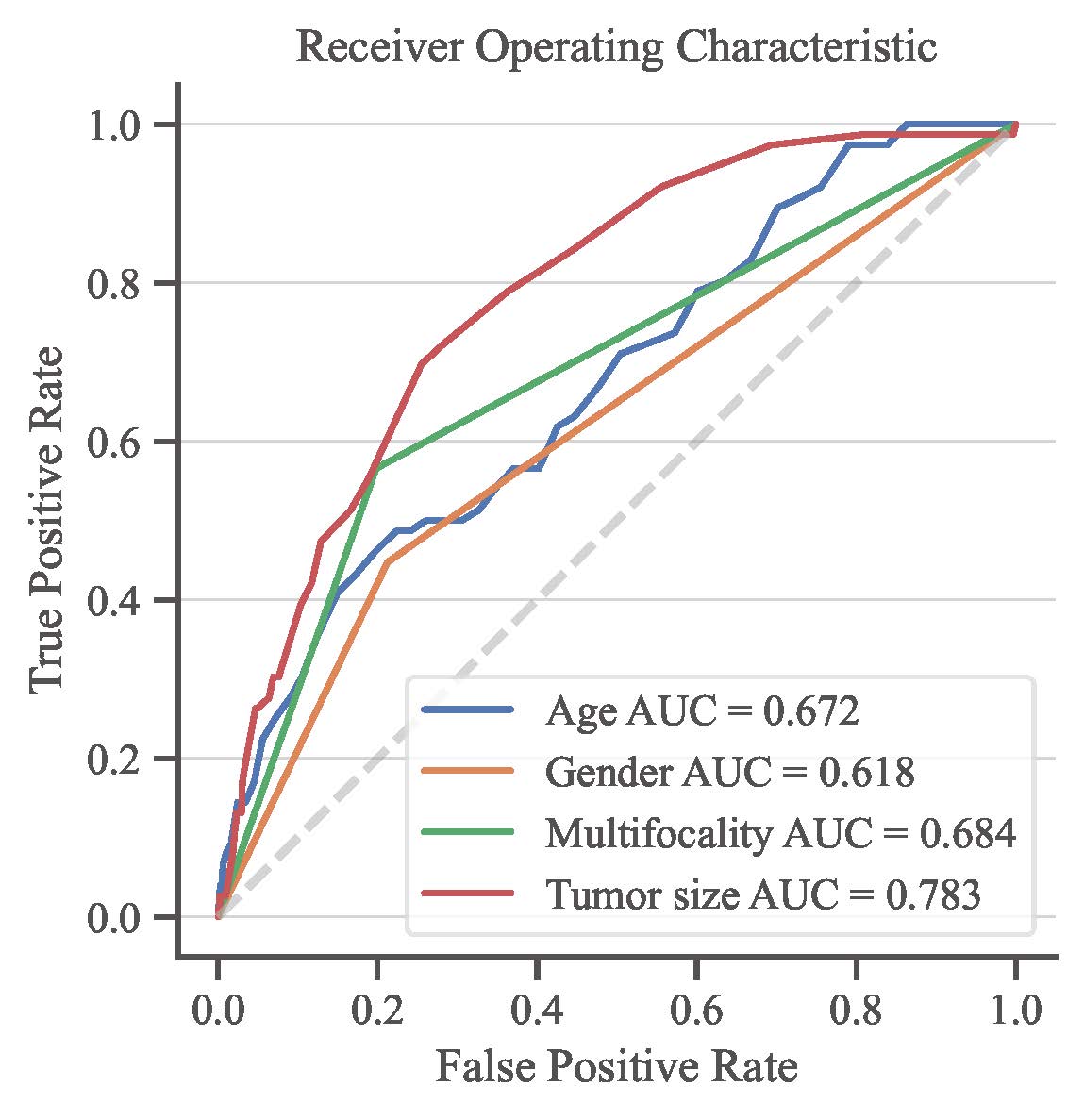 |  |

Supplementary Figure 2: ROC analysis of individual variables including gender, age, multifocality, and tumor size across the Training Set (A), Validation Set (B), and Test Set (C)

Note: ROC, Receiver Operating Characteristic

| Supplementary Table 3: Complete-case Analysis of Single-factor and Multi-factor Logistic Regression for PTC with hv-CLNM (*n*=1609) | | | | | |
| --- | --- | --- | --- | --- | --- |
|  |  | Single-factor | | Multiple-factor | |
|  |  | OR (95%CI) | *p* | OR (95%CI) | *p* |
| Gender | Female | (reference) |  | (reference) |  |
|  | Male | 3.60 (2.19-5.92) | <.001 | 2.67 (1.55-4.59) | <.001 |
| Age(y) |  | 0.96 (0.94-0.98) | <.001 | 0.96 (0.94-0.98) | <.001 |
| Location | Right | (reference) |  | - |  |
|  | Left | 1.44 (0.83-2.49) | .196 | - |  |
|  | Bilateral | 5.62 (2.78-11.39) | <.001 | - |  |
|  | Isthmus | 0.00 (0.00-Inf) | .979 | - |  |
| Multifocality | No | (reference) |  | (reference) |  |
|  | Yes | 4.37 (2.46-7.74) | <.001 | 4.65 (2.42-8.94) | <.001 |
| Size(mm) |  | 1.10 (1.08-1.13) | <.001 | 1.09 (1.06-1.12) | <.001 |
| HT | Negative | (reference) |  | - |  |
|  | Positive | 0.45 (0.19-1.06) | .068 | - |  |
| ETE | Negative | (reference) |  | (reference) |  |
|  | Positive | 1.93 (1.16-3.20) | .011 | 1.80 (1.04-3.11) | .036 |
| PTC, papillary thyroid carcinoma; CI, confidence interval; HT, Hashimoto thyroiditis; hv-CLNM, high-volume central lymph node metastasis; ETE, extrathyroidal extension | | | | | |

| Supplementary Table 4: Comparison of ETE Model and Non-ETE Model for Predicting the Performance of hv-CLNM in PTC Patients (*n*=1609) | | | | | | | |
| --- | --- | --- | --- | --- | --- | --- | --- |
|  | AUC | Sensitivity (%) | Specificity (%) | Accuracy (%) | LR+ | LR- |  |
| Non-ETE Model | 0.821  (0.762-0.879) | 77.3  (65.2-90.9) | 81.6  (64.0-86.6) | 81.3  (64.9-86.0) | 4.198  (1.811-6.779) | 0.279  (0.544-0.105) |  |
| ETE Model | 0.833  (0.78-0.887) | 81.8  (69.7-92.4) | 78.2  (68.8-86.3) | 78.3  (69.6-85.8) | 3.752  (2.231-6.759) | 0.233  (0.441-0.088) |  |
| Note: AUC, area under the roc curve; The parentheses indicate the 95% confidence interval; LR+, Positive Likelihood Ratio; LR-, Negative Likelihood Ratio; hv-CLNM, high-volume central lymph node metastasis; ETE, extrathyroidal extension | | | | | | | |

| A  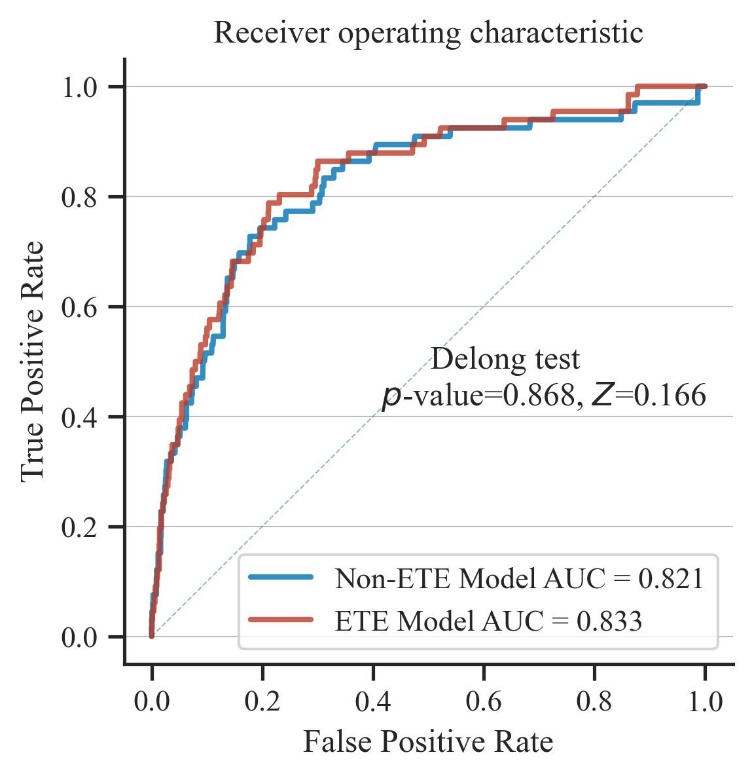 | B  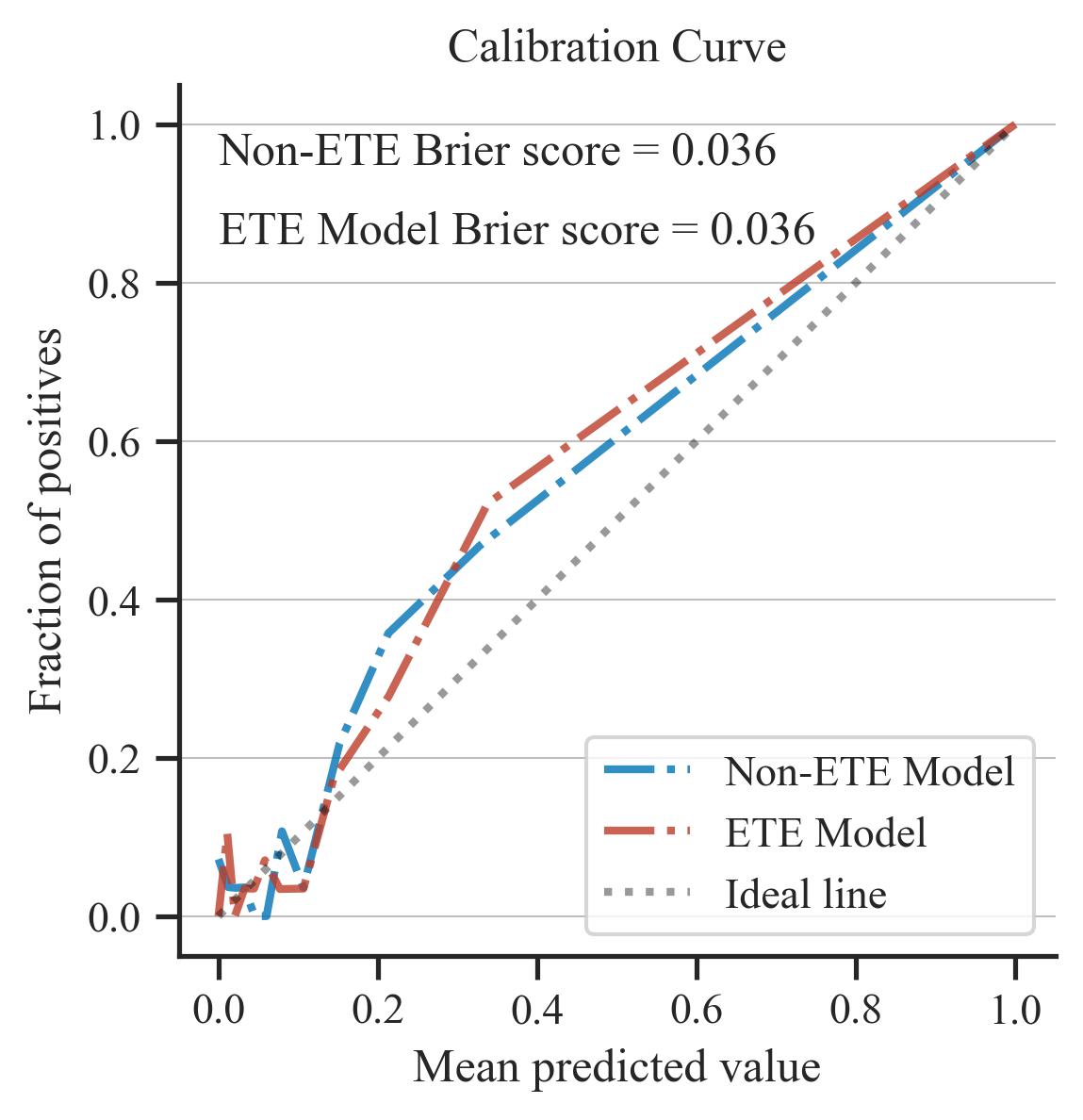 |
| --- | --- |

Supplementary Figure 3, ROC and Calibration Curves for Complete-case Analysis of the ETE Model and Non-ETE Model (*n*=1609)

Note: AUC, area under the curve; ROC, receiver operating characteristic curve; ETE, extrathyroidal extension
